# Unlocking the potential of big data and AI in medicine: insights from biobanking

**DOI:** 10.3389/fmed.2024.1336588

**Published:** 2024-01-31

**Authors:** Kaya Akyüz, Mónica Cano Abadía, Melanie Goisauf, Michaela Th. Mayrhofer

**Affiliations:** Department of ELSI Services and Research, BBMRI-ERIC, Graz, Austria

**Keywords:** biobanks, artificial intelligence, big data, European Health Data Space, infrastructures

## Abstract

Big data and artificial intelligence are key elements in the medical field as they are expected to improve accuracy and efficiency in diagnosis and treatment, particularly in identifying biomedically relevant patterns, facilitating progress towards individually tailored preventative and therapeutic interventions. These applications belong to current research practice that is data-intensive. While the combination of imaging, pathological, genomic, and clinical data is needed to train algorithms to realize the full potential of these technologies, biobanks often serve as crucial infrastructures for data-sharing and data flows. In this paper, we argue that the ‘data turn’ in the life sciences has increasingly re-structured major infrastructures, which often were created for biological samples and associated data, as predominantly data infrastructures. These have evolved and diversified over time in terms of tackling relevant issues such as harmonization and standardization, but also consent practices and risk assessment. In line with the datafication, an increased use of AI-based technologies marks the current developments at the forefront of the big data research in life science and medicine that engender new issues and concerns along with opportunities. At a time when secure health data environments, such as European Health Data Space, are in the making, we argue that such meta-infrastructures can benefit both from the experience and evolution of biobanking, but also the current state of affairs in AI in medicine, regarding good governance, the social aspects and practices, as well as critical thinking about data practices, which can contribute to trustworthiness of such meta-infrastructures.

## 1 Introduction

Life sciences knowledge production is increasingly structured by big data approaches, internationalization of research and closer coupling between research and applications, where biobanks comprise a major form of infrastructure in the current research ecosystems. For decades, biobanks have efficiently ensured access to biological samples and associated health data, which is being produced, collected and used in various ways, such as for medical research and public health databases as the two broad categories of population-based and clinical biobanks reflect (1). The historical development of the biobanks and their diversification over time contrast starkly with the current efforts for standardization, harmonization, integration, globalization and most significantly datafication. They have evolved from mere repositories to trusted infrastructures in sharing biomaterials and data (2), highlighting their crucial role in data-intensive research. These efforts for facilitating the movement of data materialized into platforms, infrastructures and guiding principles to enable the exchange of data that is compliant with ethical, legal and societal considerations.

With artificial intelligence (AI), renewed discussions are taking place due to the idiosyncrasies of AI, the speed and consequences of the implementation of such technologies in biobanking and other domains (3, 4). Over the last decade, the development of national and transnational biobank networks or infrastructures have made such infrastructures instrumental to international research consortia (5–7). In addition, meta data infrastructures called health data spaces are developed that have the potential to significantly transform the life sciences, medicine and healthcare. Back in December 2020, the European Commission published the roadmap for the European Health Data Space (EHDS) initiative inviting public responses and presenting a first draft in May 2022. Currently discussed in the European Council and the European Parliament, the ambitious goal remains to complete the legislative process by the end of 2023 but no later than within the current Commission’s mandate to ensure the implementation by 2025 (8). The EHDS will undoubtably transform the health sector in Europe. It remains to be seen in which form it will be realized, especially as expectations are high across various stakeholder groups, such as patient advocacy groups, researchers from academia and industry as well as policy makers (9). At the same time, infrastructures such as biobanks have a wealth of experience regarding the collection and use of health data for research purposes in an ethically and legally compliant way (10). The perspective we present here builds on the observation that many biobanks are already going through a transformation in becoming bio(data)banks and are entangled in trials of various data practices that can inform both the debates around AI’s use in life sciences and health research and emerging meta infrastructures considering developments, such as EU’s upcoming Artificial Intelligence Act. Although there has been a provisional agreement as of December 9^th^, 2023, among negotiators from EU’s Parliament and Council, the legal text will be implemented when the two institutions provide their approval and, if so, with its risk-based categorization and the accompanying requirements, the AI Act may have an impact on many aspects of AI’s use in health research and applications, such as on data governance, explainability, requirements, practicing human-in-the-loop among others with potential effect also on the EHDS (11). In light of these recent developments, we argue that it is timely to look back at the practice of biobanking, especially the so-called data turn, and the current momentum in biobanking and medicine regarding AI and its implementation into research and technology, for insights on health data spaces and their development.

## 2 Data turn in life sciences: biobanks as data infrastructures

Biomedical research has become increasingly data-intensive and undergone a process of datafication (12). Central to this datafication are biobanks. As infrastructures, they can be characterized as vital entities in organizing practices, as embedded in other structures, social arrangements and technologies (13). In this capacity, biobanks support medical innovation, such as personalized medicine and genomic research, with scholars noting the molecularization and computerization sustaining both (14, 15).

The molecularization and data turn in the focus of biobank research in the last two decades deserves more attention. For instance, infrastructures have been created that gather genetic data from commercial and clinical sources, enabling population-based genetics research to be conducted (16). The outcome of such research, especially in genomics, raises hopes with a better understanding of the genetic bases of health conditions such as coronary artery disease, ideally based on diverse populations (17). However, the genomic data and infrastructures raise also concerns, especially regarding phenomena, such as sexual orientation, which received renewed attention in the search for a genetic basis (18) and also harbor emerging risks that are radically different than the previous ones due to intensive datafication, for instance, risks of genomic identifiability (19).

The existence of efforts towards standardization and interoperability in biobanking as reflected in the acronyms SPREC (20), BRISQ (21), MIABIS (22, 23) and others show the centrality of these notions for the data turn, but also harmonization regarding samples, technical infrastructures and practices. The relevant research contributes to developments such as specific algorithms for post-analytical use, which may bridge the differences between distinct types of blood samples originally stored for different uses (24, 25). Such developments are especially salient considering that biobanks are not independent of the broader infrastructures of medicine and healthcare. From disease categorization to defining and standardizing biomarkers at a time wearable devices, sensors and emerging forms of data are increasingly being embedded into entire ecosystems often in the digital (26), the existing samples and data with different conditions of collection, annotation, consent status and storage, as well as variations across institutions are still part of the picture. Biobanks are expanding with both typical samples and data (e.g., blood, BMI) and further kinds (e.g., epigenetic, microbiome, etc.) being integrated and standardized, expanding the data in both dimensions of volume and diversity.

In attempts towards datafication, practices around samples such as in pathology are also being transformed, exemplified by “digital pathology” where whole slide images that are once created may decrease the need to store samples or increase the findability by turning images into data collected (27). Scholars observe along a trend of consolidation emergence of virtual biobanks brings together resources from multiple biobanks (28, 29), though such cataloging examples also include efforts of broader research infrastructures, such as BBMRI-ERIC (30). Similarly, in the genomics world, efforts to standardize and make genomic data accessible such as summary statistics of genome-wide association studies is picking up pace (31, 32) as well as the development of trusted research environments despite critique (33) with specific tools, such as DataSHIELD (34).

## 3 AI in medicine and new beginnings for biobanking

Large amounts of data are needed to advance biomedical knowledge generation as well as big data analytics and new data-driven technologies in AI. While the history of AI in medicine goes back half a century with the initiation of computational tools and technical infrastructures as well as events devoted to the topic (35), it has gained pronounced attention and applicability in recent years in line with its intensive use in other domains. Medical AI is seen as a promising innovation for uses such as screening, diagnosis, risk assessment, clinical decision-making, management planning, and precision medicine, with available tools ranging from chatbots to clinical decision support (36). The hope is that AI systems will reduce human bias and improve performance, as has been demonstrated in certain areas such as radiology (37), by improving accuracy in medical image analysis and easing the workload in screening (38), or for AI-driven polygenic risk scores (PRS) which may enable greater accuracy, performance and prediction (39). AI can also bring improvements when it comes to clinical measurements (40), interpretation of tests (41), decision making for intensive care unit admission (42), or embryo implantation (43), among others. However, it is important to note that AI is not a one-size-fits-all solution, and its benefits may not be realized in every application.

The development and implementation of medical AI involves numerous key challenges. First, AI is data hungry. Large amounts of data are needed to train AI and access to these data is challenging for technical, legal, and practical reasons, along with emerging issues regarding computational power and infrastructures and alternatives such as federated learning, which bring their own challenges and opportunities (44). One salient challenge in this respect relates to the tradeoff between data access and data privacy, the resolution of which necessitates bottom-up, democratic and engaging processes (3) in consideration of commitment for findable, accessible, interoperable and reusable data as often referred to with the acronym FAIR (45) and further FAIR principles (e.g., https://www.go-fair.org/fair-principles/). Second, despite the immense potential benefits, the risks revolve around perpetuation or even amplification of societal inequality and injustices due to potentially biased datasets as well as certain data practices (46). Third, practitioners require practical recommendations for applying AI (47). Furthermore, patients’ preference for human agents or human supervision, possible strain between patients and treating physicians, especially in relation to privacy, data security and potential vulnerabilities related to AI tools need attention as do the implementation of guidelines and frameworks to ensure bioethical principles [e.g., (48)] are upheld and monitored (49). These call for engagement of multiple stakeholders in the resolution of ethical and legal issues, sharing similarities with biobanking, though at a different scale.

Biobanks, as key entities for providing access to large amounts of high-quality data, are central to the development of new data-based technologies such as AI. Similar to AI in medicine, the early developments in the use of AI in biobanking often focus on biobank participants’ health conditions as reviewed elsewhere (50). These include developments such as, identifying and categorizing Alzheimer’s disease patients (51), calculating risks scores for conditions such as age-related macular degeneration (52) or cardiovascular diseases (53), aiding in classification of disease subtypes (54) as well as providing predictions at individual level for COVID-19 (55, 56) or potential conditions due to therapeutic agents such as aromatase inhibitor-related arthralgia (57). However, biobanks are not merely support structures for healthcare or repositories for medical data. Biobanks have the potential to handle the data turn as they pursue data-driven practices in a standardized, industrialized manner (58). As research infrastructures, biobanks, may benefit from AI in the collection of biological samples and data, such as analysis of the scholarly literature for development of criteria for sampling, analysis, interpretation, data extraction, even engagements with biobank participants, from consent process to research process; however, AI can also contribute to purely *managerial tasks* including storage space optimization or *upstream research processes*, such as suggesting samples and data for research proposals based on content and methods, as well as *downstream research evaluation*, assessing the “value” of samples and data based on the scholarly literature (59). AI’s potential impact on biobanking may also include possible increases in the use of biobank samples and data, thus contributing to sustainability and speed of research as well as aiding biobanks in identification and recruitment of participants, training, annotation of samples and data, increasing interoperability, visibility, and access (60).

AI is central to the idea of “biobanks for the future” (61) though challenges in implementation of AI in biobanking range from difficulties aligning standards not only across data in the long run, but also samples, workflows, ethics management, legal and governance-related aspects, from transparency to informed consent (28) as well as justice, both epistemically and ethically (14). There are efforts such as workshops or collections of best practices to increase the “readiness” of these infrastructures for AI (60) with calls, checklists, tools and frameworks for ethical use of AI in medicine/biobanking (47, 62). New and alternative forms of governance are needed for a new form of biobanking that revolves around big data considering the increasing widening of the scope of data from social media to devices capturing bodily function, resulting in streams of data over *time* and analytical capacity over *space* (63). Biobanks’ positioning at the in practice often gray intersection of healthcare and research can inform the discussions on health data spaces, in light of the recent developments.

## 4 Discussion

The ways in which risks are approached in biobanking and the normative arguments regarding how they should, such as future-proofing the governance of biobanks (64) and adaptive risk governance (65), suggest biobanking may be helpful in identifying key questions medical AI and health data spaces are facing from informed consent, representation in datasets, to risks associated with data protection and responsibility. While acknowledging the digital divide and its consequences, the increased ability of participants to follow and engage with biobanking and healthcare infrastructures are leading to reconfigurations of “traditional boundaries between the public domain (healthcare systems, medical research, and clinical practice) and the private one (patients and citizens)” which necessitate new approaches to fostering trust (63). Health data spaces bring such observations to a new level.

Trust and trustworthiness have become keywords that are often attached to how AI should be, with limited discussion of what this entails. Despite the burgeoning literature on ethics of AI in medicine, three areas relevant for trust are problematic (46): limited analytical accuracy and conceptual slippages, inadequate analysis of the contexts in which medical AI tools are embedded, and scarcity of interdisciplinary approaches. Considering trust central to societal functioning as “a fundamental principle for interpersonal interactions” (66), it cannot be considered unidirectional. Rather, it needs to be understood as a complex, situated, context-dependent, and relational concept that involves several trustor/trustee relationships, such as trust in persons (e.g., scientists who trust each other, patients who trust scientists and clinicians), technology, and institutions (67, 68). Trust or more precisely trusting relationships are fragile and require continuous work, which means that they need to be actively established and sustained. In this sense, we see three main considerations from biobanking – a domain that should be built on trust – that can contribute to better medical AI and health data spaces.

*Regulations may provide guidance, but good governance is an active process that comprises more than following regulations*. Efforts towards regulating and guiding AI have been abundant with ‘AI Ethics’ becoming a buzzword (69, 70) along with the legal frameworks such as the proposed Artificial Intelligence Act of the EU (11). Considering international standards, overseeing organizations, national legislations, as well as practices, from engaging participants to consents, biobanks have accumulated over decades experiences related to intensified transnational data sharing, international collaborations, including public-private partnerships, access to and reuse of data, and efforts to harmonize data, ethical/legal standards and societal aspects. Hence, biobanking incorporates knowledge of the “ethics work” that is an integral part of data flows (71) and necessitates thinking critically about potential issues that go beyond individual institutions, such as identifiability risks in a datafied world both in regards to genomic (19) and medical imaging data (72). Thus, necessary good governance involves more than procedure-following.

*Infrastructures are not merely technical,* i.e.*, buildings, data repositories, but also social – involving practices*. A recent study (73) with biobank professionals and experts indicates that expectations towards biobanks in view of data processing are going beyond their status as repositories. They see biobanks in a more active role when it comes to providing information and communicating and engaging with biobanks participants and point to the need to improve consent procedures and the role of biobanks in sharing samples and data with industry partners and different countries. Considering that participants are the origin of the data, as key stakeholders they should be involved in the development and governance, just as staff in biobanks should be included (74). Decades of biobanking show that *the concerns of citizens cannot be ignored*. In the case of AI in health, these not only relate to the general concerns regarding AI. On the contrary, as suggested by the PRS and AI, ethical, legal and societal issues necessitate a layered understanding due to increasing complexity bringing new relevance to concepts such as explainability and interpretability, both for the users and the broader society (39). Considering the drivers of AI in medicine, such as identification and management of potential patients that can be “high-risk” but also “high-cost” (75), the developments may not benefit individuals who may otherwise develop conditions that are harder to treat or identify and manage emerging outbreaks in real-time, and such AI tools may cause further burdens on the individuals. These necessitate societal debates and empowering citizens, including involving potential non-users, as part of bringing infrastructures to life (76).

*Not only are data not always perfect due to inherent finite categorization of potentially infinite diversity, but their capacity to represent should always be continuously problematized*. Against the biobanking professionals’ concerns, the tendency to see biobanks as data repositories and medicine as increasingly digital (27, 63) can result in a false sense of security in the imaginary of increasing data interoperability and connectedness at the peril of ignoring what D’Ignazio and Klein (77) rightly note the existence of “problems that cannot be represented—or addressed—by data alone” (p. 10). Risks accompany the opportunities in a datafied world. The existence of data should not automatically lead to testing of any potential association and scholars have been trying to identify ways of coping with such issues of reproducibility, e.g., for PRS (78, 79). In this regard, the “curse of dimensionality” in biobanking due to multitude of secondary data even in cases of low sample sizes, can also be seen as an opportunity to think outside of the box to overcome issues even in smaller sample size situations (80). Furthermore, AI may also exacerbate the existing big data issues that are yet to be resolved. While the uses may relate to privacy with unintended access to data from patient implants, sensors and other devices that collect and transfer multiple forms of data, they may also lead to spurious correlations and false positives, tacit assumptions regarding individual behavior based on limited data, sampling issues due to replacement of traditional ways of data collection as well as resulting in injustices due to resource mismanagement and allocation, especially in case of public health issues (81). With health data spaces, these issues will likely need more attention.

Projectified ways of health infrastructuring often restrict the outcome in many ways, through visions and expectations for whom and which purposes the infrastructure is to be developed even in cases where the aim is to involve stakeholders in co-creation processes (76). In this paper we have shown the wealth of knowledge generated through the use of AI in medicine and the evolution of biobanking. We argue, when taken into account, these can positively impact the future European Health Data Space, but also similar establishments, giving power to the citizen, strengthening governance, breaking down potential silos and contributing to trustworthiness of such meta-infrastructures.

## Data availability statement

The original contributions presented in the study are included in the article/supplementary material, further inquiries can be directed to the corresponding author.

## Author contributions

KA: Conceptualization, Writing – original draft, Writing – review & editing. MA: Conceptualization, Writing – original draft, Writing – review & editing. MG: Conceptualization, Writing – original draft, Writing – review & editing. MM: Writing – original draft, Writing – review & editing.
